# Second primary mediastinal grey-zone lymphoma following treatment for classical Hodgkin lymphoma: a rare case report

**DOI:** 10.3389/fonc.2026.1913997

**Published:** 2026-08-12

**Authors:** Yuan Tan, Liqin Lai, Ting Huang, Wei Wang, Xiaoyu Han

**Affiliations:** Department of Pathology, Tongde Hospital of Zhejiang Province, Hangzhou, China

**Keywords:** classical Hodgkin lymphoma, mediastinal gray-zone lymphoma, primary mediastinal large B-cell lymphoma, second malignant neoplasm, therapy

## Abstract

**Background:**

Gray-zone lymphoma (GZL) is a rare and biologically distinctive B-cell lymphoma that exhibits overlapping clinical and pathological features between classical Hodgkin lymphoma (cHL) and mediastinal large B-cell lymphoma (PMBL). Mediastinal Gray-zone lymphoma (MGZL) occurring as a second primary malignancy following treatment for cHL is exceedingly rare, and both its pathogenesis and optimal clinical management remain poorly characterized.

**Case presentation:**

A 22-year-old woman diagnosed with cHL in 2020 achieved complete remission after ABVD chemotherapy. Five years later, a new mediastinal mass was identified during routine follow-up. Excisional biopsy via video-assisted thoracoscopic surgery (VATS), together with comprehensive immunohistochemical and molecular analyses, confirmed a diagnosis of secondary MGZL. The patient subsequently underwent salvage chemotherapy followed by autologous stem-cell transplantation, achieving clinical remission.

**Conclusion:**

In long-term survivors of cHL, the emergence of a new mediastinal mass necessitates repeat biopsy and precise pathological reassessment rather than presumption of relapse. Second primary lymphomas, such as MGZL, require individualized treatment strategies.

## Introduction

Hodgkin lymphoma (HL) is a clonal B-cell malignancy accounting for approximately 10% of all lymphoid neoplasms, with classical Hodgkin lymphoma (cHL) representing the most common subtype. Histologically, cHL is characterized by the presence of scarce malignant Hodgkin and Reed-Sternberg (HRS) cells within a prominent reactive inflammatory cells (1). Therapeutic outcomes for cHL have improved markedly, with current cure rates reaching 80%-90%. These advances are largely attributable to modern chemoradiotherapy regimens (such as ABVD and BEACOPP), alongside continuous refinements in targeted agents, immunotherapy, and transplantation techniques. These advancements enable most patients to achieve long-term remission or even clinical cure (2). However, this substantial improvement in survival has unveiled a broad spectrum of therapy-related late toxicities, most notably second malignant neoplasms (SMNs), as well as cardiovascular, pulmonary, and infectious complications (3). Gray-zone lymphoma (GZL) is a rare, aggressive B-cell neoplasm characterized by pathologic features that overlap between two well-defined lymphoma entities, defying classification into either category. Mediastinal gray-zone lymphoma (MGZL) represents the most well-characterized and clinically relevant subtype of GZL. It is defined by its mediastinal location and exhibits overlapping clinical, morphologic, and immunophenotypic features of primary mediastinal large B-cell lymphoma (PMBL) and cHL, particularly the nodular sclerosis subtype (4). Typically manifesting as a bulky anterior mediastinal mass in adolescents or young adults, MGZL is exceedingly rare as a SMN in long-term survivors of cHL. The overlap between MGZL and both cHL and PMBL confers a substantial risk of misdiagnosis, and no single specific biomarker currently exists to establish the diagnosis definitively. Consequently, the diagnosis relies on an integrated assessment of a comprehensive immunohistochemical panel and the systematic exclusion of both cHL and PMBL. Nevertheless, accurate recognition of MGZL is clinically critical, as its treatment strategies and prognosis differ substantially from those of cHL and PMBL. Notably, MGZL typically exhibits a more aggressive clinical course with inferior outcomes compared with either entity. We herein present an exceedingly rare and instructive case of a patient with cHL who MGZL developed as a second primary malignancy, at the original site five years after achieving complete remission. A comprehensive analysis of this case underscores the critical importance of long-term surveillance, repeat biopsy, and integrated multidisciplinary evaluation.

## Case presentation

### Clinical findings

**Figure 1:**
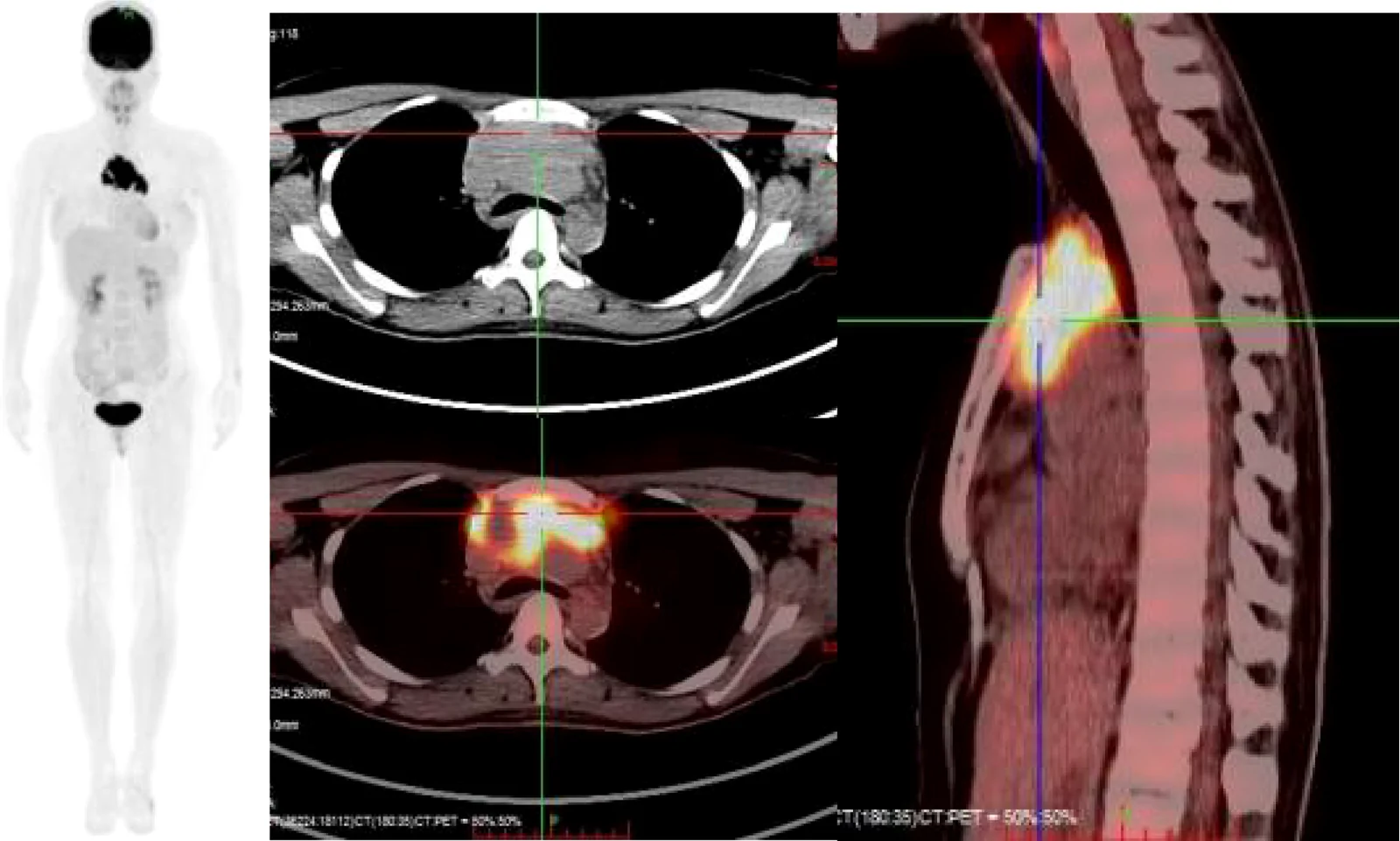
Whole-body MIP image demonstrates intense FDG uptake (SUVmax 20.0) localized to the anterior mediastinum, with physiologic background activity elsewhere. Axial PET-CT fusion image shows a 6.8 × 3.4 cm heterogeneous hypermetabolic mass encasing adjacent mediastinal vessels.

**Figure 2:**
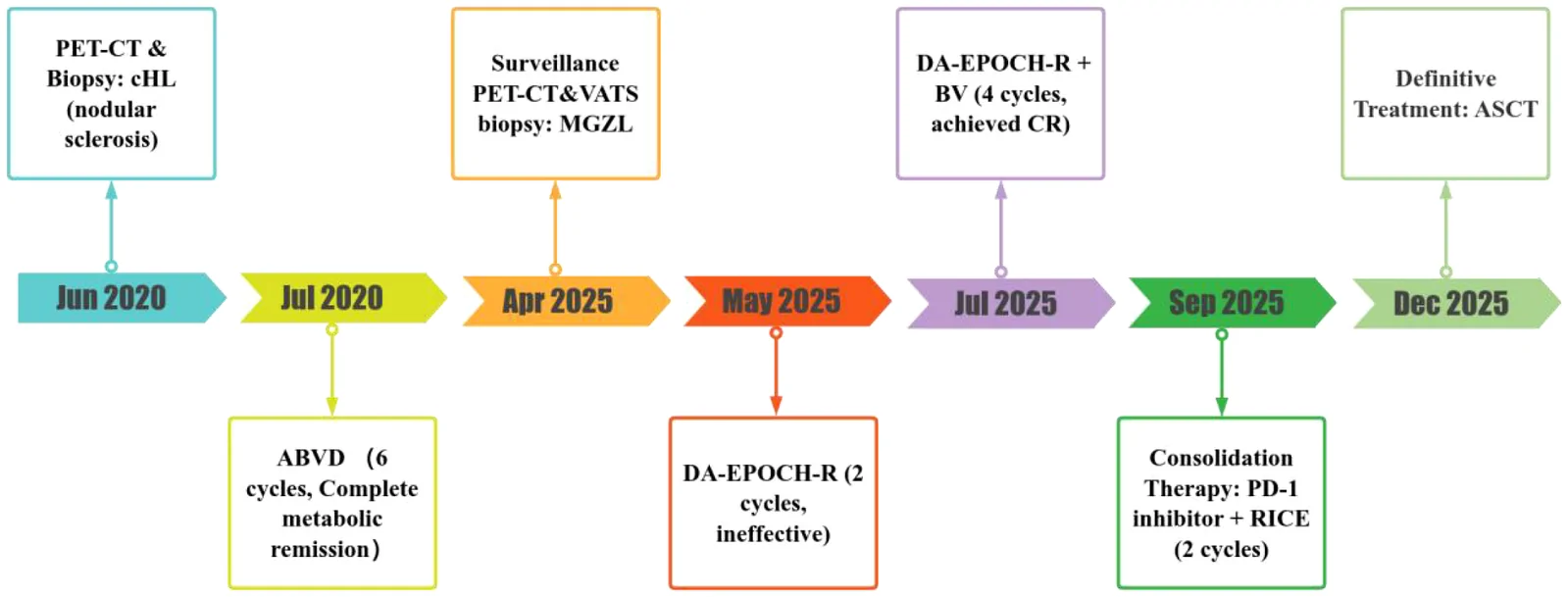
Simplified timeline of clinical course and treatment.

In June 2020, a 22-year-old woman presented with unexplained dyspnoea and a self-palpated cervical mass. PET- CT revealed multiple FDG-avid lymph nodes involving the bilateral cervical chains, supraclavicular regions, and superior mediastinum. Baseline imaging from the initial diagnostic work-up (2020) had been performed at an external institution and could not be retrieved despite multiple requests. Histopathological examination of a right cervical lymph node biopsy confirmed nodular sclerosis cHL. The patient’s immediate family members were healthy, with no family history of genetic disorders. Treatment with the ABVD regimen was initiated in July 2020. Following six cycles of chemotherapy, the patient achieved complete metabolic remission, which was sustained on subsequent surveillance imaging. Surveillance PET-CT in April 2025 disclosed a superior mediastinal mass highly suggestive of lymphoma recurrence (**Figure 1**). Video-assisted thoracoscopic surgery (VATS) biopsy confirmed MGZL with infiltration into adjacent lung parenchyma. Bone marrow aspiration showed no morphologic or immunophenotypic evidence of lymphomatous involvement. Subsequently, the patient received two cycles of DA-EPOCH-R chemotherapy in May, with documented disease progression. In July, treatment was switched to the DA-EPOCH-R plus brentuximab vedotin (BV) regimen. After four cycles of this combined therapy, follow-up PET-CT demonstrated a complete response (CR). To achieve deeper remission prior to autologous stem-cell transplantation (ASCT),the patient received two cycles of PD-1 inhibitor plus RICE in September 2025, followed by successful ASCT in December 2025 (**Figure 2**). At the time of writing, the patient remains in continuous complete remission during the 6 months post-transplantation and continues under active surveillance.

### Pathological findings

**Figure 3:**
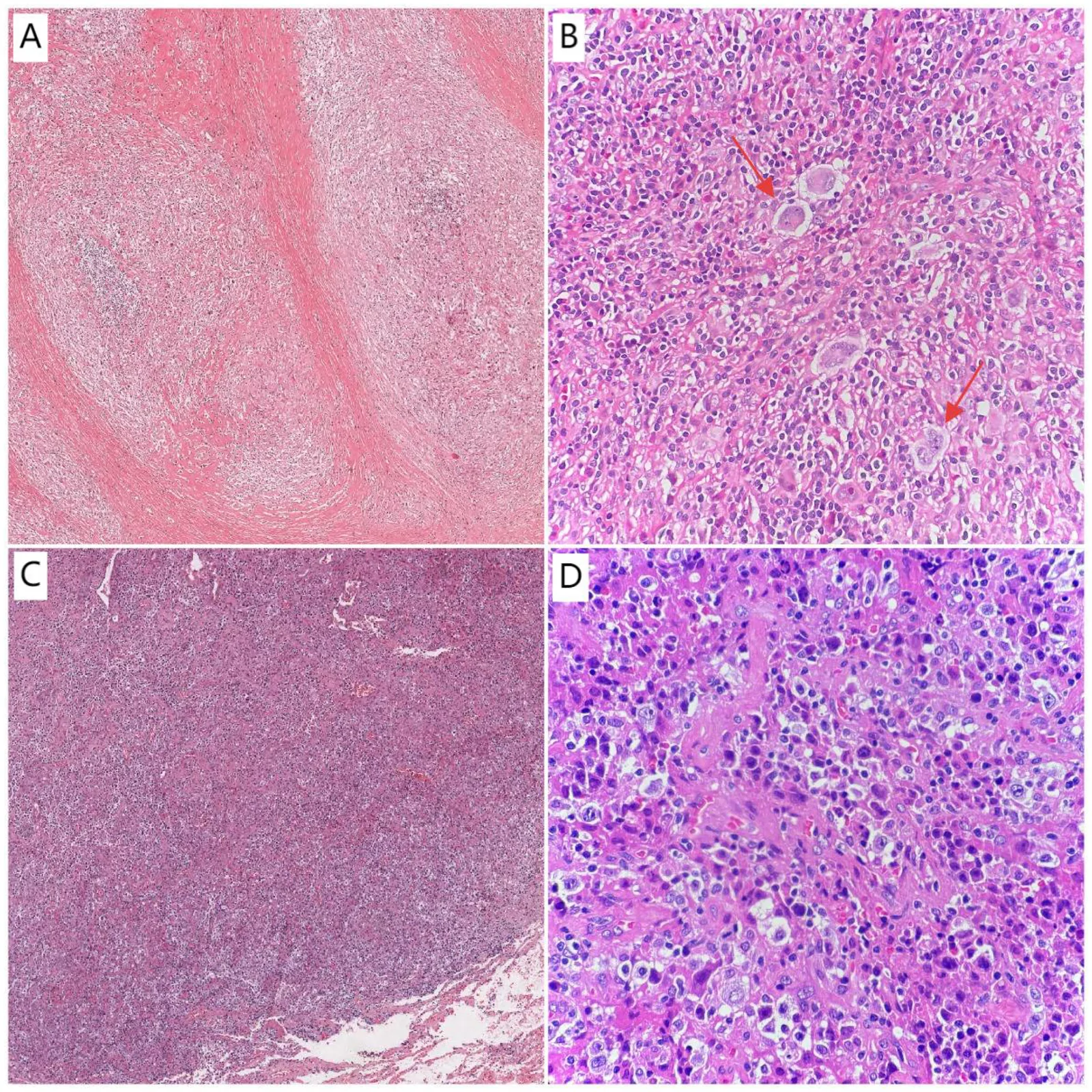
**(A, B)** Primary biopsy showing cHL, nodular sclerosis subtype: **(A)** normal lymph nodal architecture is effaced, fibrous bands partition the parenchyma into variably sized nodules (H&E, ×40), **(B)** typical HRS cells are present within an inflammatory background (H&E, ×400). **(C, D)** Secondary biopsy demonstrating MGZL: **(C)** The mediastinal tissue is diffusely infiltrated by neoplastic cells separated by irregular collagenous trabeculae, forming sheets or ill-defined nodules (H&E, ×40), **(D)** Tumor cells exhibit diffuse, cohesive growth; they possess large, hyperchromatic nuclei and abundant pale to clear cytoplasm (H&E, ×400).

**Figure 4:**
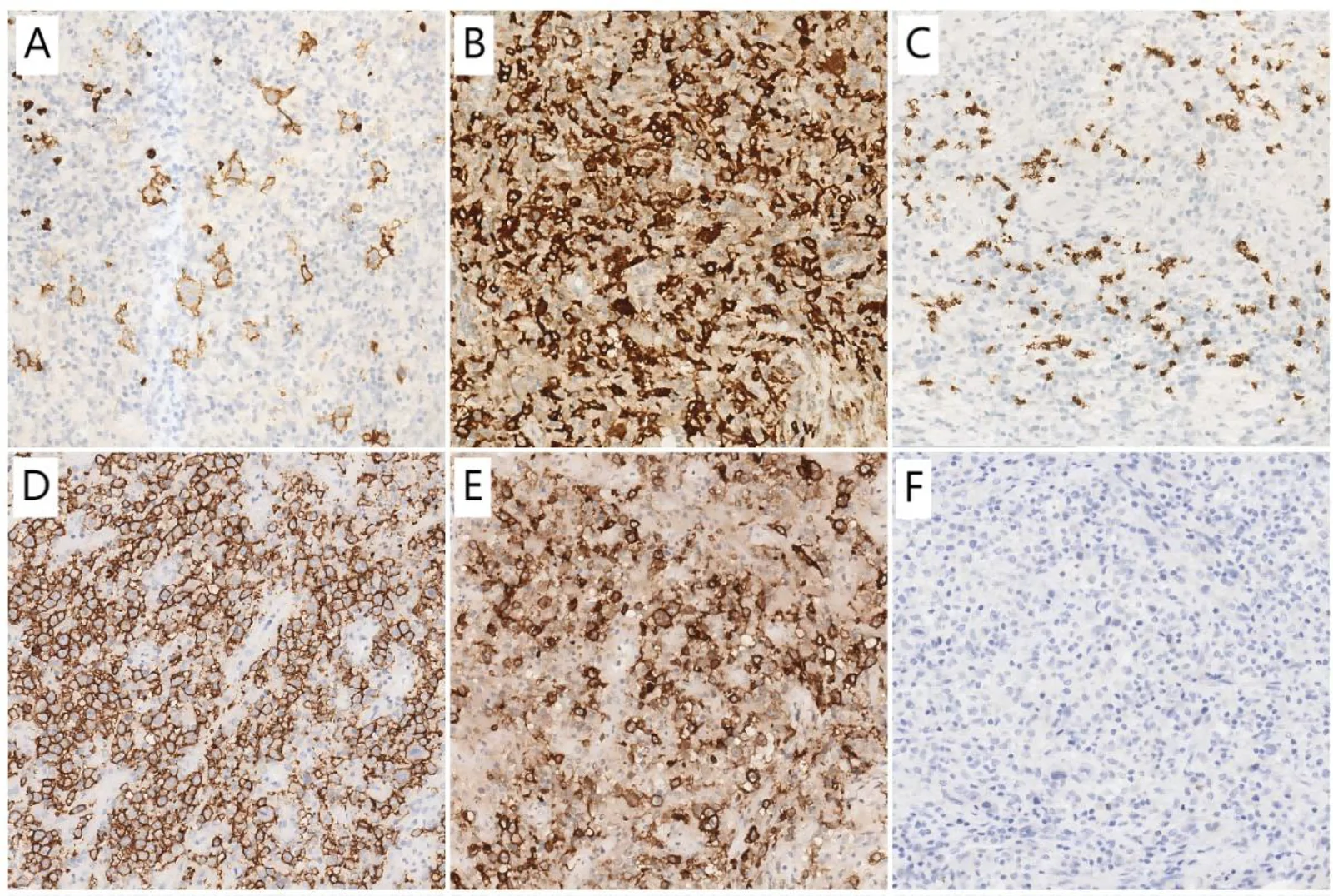
**(A–C)** Initial biopsy of cHL, nodular sclerosis subtype (all images: IHC, ×200): **(A)** CD20 immunostaining demonstrates weak positivity in R-S cells; **(B)** CD30 reveals strong positivity; **(C)** CD15 is focally positive. **(D–F)** Second biopsy of MGZL: **(D)** CD20 shows strong positivity in tumor cells; **(E)** CD30 is strongly positive; **(F)** CD15 is negative.

**Figure 5:**
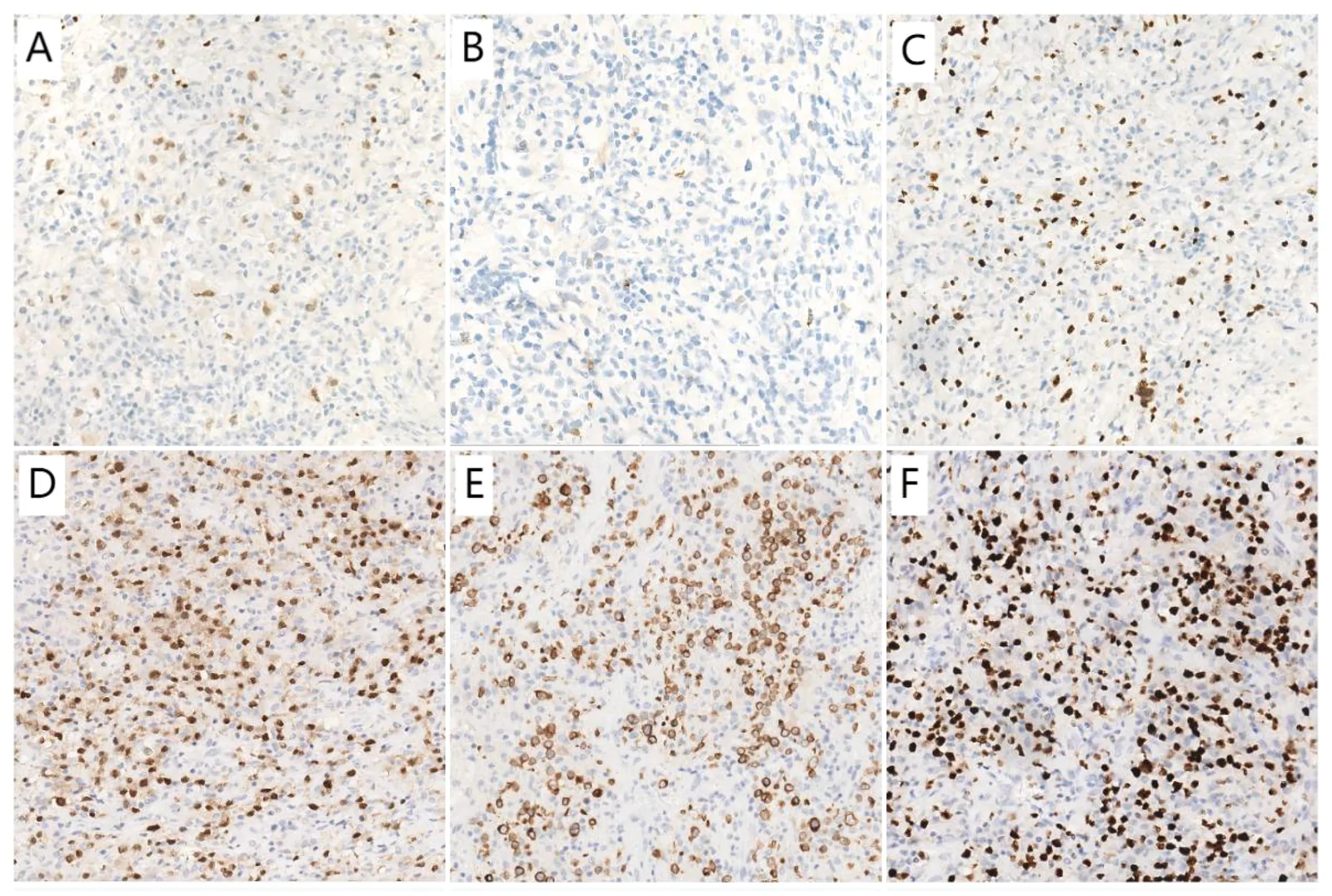
**(A–C)** Initial biopsy of cHL, nodular sclerosis subtype (all images: IHC, ×200): **(A)** PAX-5 immunostaining shows weak positivity; **(B)** CD79a is negative; **(C)** Ki-67 proliferation index is approximately 30%. **(D–F)** Second biopsy of MGZL: **(D)** PAX-5 is positive in tumor cells; **(E)** CD79a is positive; **(F)** Ki-67 proliferation index is approximately 80%.

In the present case, all biopsy slides were independently reviewed by senior hematopathologists. The initial right cervical lymph-node biopsy obtained in 2020 revealed complete effacement of the normal nodal architecture with marked capsular thickening. Broad collagen bands traversed the parenchyma, subdividing it into variably sized nodules and creating the characteristic nodular sclerosis pattern (**Figure 3A**). Scattered within this inflammatory milieu were mononuclear Hodgkin cells and classic binucleated Reed-Sternberg cells. The latter displayed abundant, slightly eosinophilic cytoplasm; large, mirror-image nuclei with sharply defined nuclear membranes; and centrally placed, prominent, round, eosinophilic macronucleoli imparting an “owl-eye appearance” (**Figure 3B**). Immunohistochemical profile: CD3 (+, T cells), CD20 (+, weak) (**Figure 4A**), CD30 (+) (**Figure 4B**), CD21 (+, follicular dendritic-cell meshwork), CD23 (+), CD163 (+, histiocytes), CD15 (+, focal) (**Figure 4C**), CD5 (+, T cells), PAX-5 (+, weak) (**Figure 5A**), PCK (–), Oct-2 (–), Bob-1 (+), Bcl-2 (+), Bcl-6 (–), CD10 (–), ALK (–), CD79a (–) (**Figure 5B**), Ki-67 (+, 30%) (**Figure 5C**), LCA (–). *In situ* hybridization for EBV-encoded RNA (EBER) was negative. Collectively, these morphological and immunophenotypic findings supported a diagnosis of cHL, nodular sclerosis subtype.

Histopathologic examination of the second mediastinal biopsy (2025) revealed complete effacement by a diffuse neoplastic infiltrate. Broad, variably thick collagen bands traversed the tumor, partitioning neoplastic cells into irregular nodules and sheets and creating a coarse-trabecular sclerotic pattern lacking the well-circumscribed fibrotic rims typical of nodular sclerosis cHL (**Figure 3C**). The inflammatory background was notably sparse, lacking significant eosinophil or plasma cell infiltration. The neoplastic cells displayed a diffuse, confluent growth pattern; they were large, with abundant pale to clear cytoplasm, and possessed round to irregular nuclei containing single or multiple small nucleoli. Occasional Reed-Sternberg-like multinucleated giant cells were observed (**Figure 3D**). Immunohistochemistry yielded the following results: CD10 (–), CD163 (+, histiocytes), CD20 (+) (**Figure 4D**), CD21 (–), CD3 (+, small lymphocytes), CD30 (+) (**Figure 4E**), CD15 (–) (**Figure 4F**), CD5 (+, small lymphocytes), PAX-5 (+) (**Figure 5D**), CD79a (+) (**Figure 5E**), Bcl-2 (+), Bcl-6 (+, 30% focal), ALK (–), Oct-2 (+), Bob-1 (+), LCA (+), Ki-67 (+, 80%) (**Figure 5F**), c-Myc (+, 35%), TTF-1 (–), S-100 (–). EBER *in situ* hybridization was negative. Fluorescence *in situ* hybridization (FISH) revealed no *TP53* deletion and no rearrangements involving *BCL2*, *BCL6*, or *MYC*. Next-generation sequencing (NGS) using a 218-gene lymphoma panel identified 27 genetic alterations, of which 6 were deemed to be of definite or potential clinical significance. Sequencing analysis revealed STAT6 p.D419N mutation (a recurrent activating alteration in PMBL and MGZL), a loss-of-function splice-site mutation in CIITA (a molecular signature of PMBL/MGZL), and concurrent inactivating alterations in TNFAIP3 and B2M. Copy number variation (CNV) analysis of a 24-gene panel (encompassing *PD-L1*, *JAK2*, *REL*, *BCL2*, *MYC*, *CDKN2A*, and *TP53*) revealed no significant copy number alterations above the reporting threshold. This mutational profile is consistent with immune evasion mechanisms and constitutive activation of NF-κB and JAK-STAT signaling pathways, features characteristic of mediastinal lymphomas. Integrating the morphological, immunophenotypic and molecular findings, the diagnosis was consistent with MGZL, as defined in the World Health Organization Classification of Hematopoietic and Lymphoid Tissues, 5th edition (2022).

## Discussion

In long-term survivors of cHL, a newly emerging mediastinal mass is often intuitively regarded as cHL relapse. However, as illustrated by the present case—in which the patient developed MGZL following cHL—such assumptions risk misdiagnosis and inappropriate therapeutic planning. We therefore strongly recommend that any new or enlarging mediastinal lesion arising within or adjacent to the previous field, regardless of latency, undergo repeat adequate tissue biopsy, preferably via VATS resection or multiple core needle biopsies. The diagnostic challenge of MGZL primarily stems from its inherent “intermediate” biology: neoplastic cells may simultaneously exhibit HRS-like morphology typical of cHL and large B-cell morphology characteristic of PMBL. Consequently, the immunophenotype fully satisfies neither cHL nor PMBL criteria. Morphologically, the mark of cHL is scattered HRS cells within a dense inflammatory background composed of lymphocytes, eosinophils, and other immune cells. Immunophenotypically, characteristic markers include CD30 and CD15 positivity, while B-cell markers (e.g., CD20, CD79a, OCT-2 and BOB.1) are typically weak or absent (5). The initial biopsy was consistent with these findings. In contrast, PMBL is characterized by sheets of medium-to large-sized B-cells with abundant clear cytoplasm, which are separated by fibrotic stroma, and usually shows minimal inflammatory infiltrate. Immunophenotypically, PMBL demonstrates a full B-cell profile (with strongly positivity for B-cell markers) and is generally CD30-negative or only weakly positive (6). The hallmark of MGZL is the concurrent expression of cHL markers (CD30) and PMBL features (strong B-cell markers such as CD20). When cHL-like morphology is accompanied by prominent CD20 expression, the possibility of MGZL should be considered. When strong and diffuse nuclear staining for B-cell markers such as OCT2 and BOB.1 is also present, the diagnosis of MGZL is strongly supported. In the present case, comparison of the two biopsies provided direct and compelling evidence for a second primary MGZL rather than cHL relapse: CD15 shifted from positive to negative, CD20 from weak/focal to strong and diffuse, and CD79a and OCT2 turned from negative to positive—a profile fully consistent with MGZL.

The emergence of a morphologically distinct second lymphoma (MGZL) at the identical mediastinal site years after curative therapy for cHL supports the hypothesis of a shared progenitor cell or niche, highlighting treatment-induced oncogenic pressure as a particularly relevant contributing factor. The most compelling hypothesis posits that both tumors arise from a single, genetically vulnerable precursor B cell; the initial oncogenic hit gave rise to cHL, whereas a second, distinct genetic lesion in a residual (or never-eradicated) subclone, occurring years later, redirected differentiation toward MGZL (7). Recent deep-sequencing studies have demonstrated that cHL and MGZL originate from shared oncogenic pathways but undergo divergent evolution. Both entities exhibit constitutive JAK/STAT and NF-κB signaling, frequently driven by alterations involving SOCS1, STAT6, TNFAIP3, and copy-number gains of 9p24.1 (which harbors JAK2 and PD-L1/L2). It is hypothesized a baseline alteration in one of these pathways within the founding clone may precede the acquisition of cooperating mutations—such as CIITA fusions, enriched in both PMBL and MGZL—that subsequently determine the final histologic phenotype (8).Underlying immunodeficiency or genetic predisposition may also be present in the affected individual. The unique thymic/medullary lymphoid niche provides survival and growth signals essential for the development of both cHL and MGZL. Initial chemotherapy or radiotherapy may suppress the dominant cHL clone but does not abrogate the fundamental microenvironment—the “soil” that supports lymphomagenesis. Consequently, a persisting abnormal B cell or a newly recruited one homing to the same supportive niche may evolve into MGZL (9). The identical anatomic location of both lesions in the present case suggests that the local microenvironment exerts a “directional” influence on phenotypic switching. Crucially, DNA-damaging agents (especially alkylating agents) induce DNA cross-links, point mutations, and chromosomal aberrations, thereby activating oncogenes and generating genomic instability that favors new driver mutations (10). The MGZL focus in the previous field strongly implies therapy-associated oncogenesis; furthermore, alkylator-related secondary lymphomas usually exhibit a short latency (5-10 years), a time frame that aligns with the five-year interval observed in this case.

Given the rarity of MGZL, no consensus treatment guidelines have been established; clinical management is therefore individualized and frequently extrapolated from therapeutic algorithms for refractory cHL and PMBL. In the present case, NGS revealed a distinctive mutational profile characterized by concomitant alterations in genes involved in antigen presentation (*B2M* and *CIITA*), oncogenic signaling (*STAT6* and *TNFAIP3*), and transcriptional regulation (*MGA*), supporting the hybrid molecular nature of MGZL as an intermediate entity between cHL and PMBL. Crucially, these genomic alterations provided the biological rationale for our dose-intensified therapeutic approach. The constitutive activation of survival pathways, evidenced by a *STAT6* mutation (p.D419N, affecting the DNA-binding domain) and a *TNFAIP3* truncation (p.K91Sfs*5), indicates dependence on JAK/STAT and NF-κB signaling, respectively. These alterations justified the upfront use of dose-adjusted EPOCH-R (DA-EPOCH-R) rather than standard R-CHOP, given that dose-intensified regimens are particularly effective in PMBL-like molecular phenotypes with activated NF-κB signatures (11). Furthermore, universal CD30 expression in MGZL (shared with cHL) and the NF-κB-independent mechanism of action of BV provided a compelling rationale for adding BV following a suboptimal metabolic response to DA-EPOCH-R alone (12). The tumor exhibited bimodal loss of antigen presentation, resulting from B2M mutations (p.M1? and p.D96Rfs*2) that abrogate HLA class I and a CIITA splice-site mutation (c.3062 + 1G>A) that disables HLA class II. This loss establishes a state of “immune ignorance” (13), typically associated with aggressive disease. Paradoxically, these same defects create a dependency on alternative immune checkpoints for survival, most notably the PD-1/PD-L1 axis. PD-L1 upregulation in MGZL is frequently driven by 9p24.1 amplification, a genetic feature shared with PMBL and cHL (8). Although no copy number alterations were detected by the 24-gene CNV panel in this case, the absence of 9p24.1 amplification is noteworthy. While 9p24.1 gains are characteristic of MGZL and predictive of PD-1 inhibitor response, their absence does not preclude efficacy, as the patient’s *B2M* and *CIITA* mutations indicated an immunoevasive phenotype still amenable to checkpoint blockade. The subsequent PD-1 inhibitor plus RICE regimen served complementary roles: PD-1 blockade targeted the PD-L1-mediated immune privilege of MGZL, while RICE provided high-dose cytotoxic salvage therapy (14). Bryan L J et al. (15) demonstrated that adding PD-1 inhibitors to ICE chemotherapy in relapsed cHL achieved 86.5% CR rates without compromising stem cell mobilization. Given that MGZL shares cHL’s mediastinal predilection and frequent CD30 expression, this provides a clinical precedent for the PD-1 inhibitor plus chemotherapy backbone approach. Importantly, this regimen served as an effective bridge-to-transplant strategy to eradicate residual disease prior to ASCT. Finally, ASCT consolidation performed in December 2025 capitalized on the complete metabolic remission achieved, consistent with established guidelines (e.g., NCCN/EBMT) that recommend consolidative autologous transplantation for patients with chemotherapy-sensitive relapsed or refractory lymphoma who achieve complete or good partial responses. In summary, the mutational profile guided our multimodal therapeutic strategy of dose-intensified immunochemotherapy, checkpoint inhibition, and ASCT consolidation. This precision approach, targeting specific molecular vulnerabilities rather than relying on empirical histologic classification, achieved sustained complete remission in this challenging second-primary MGZL following cHL. To our knowledge, this represents the first reported application of genomic-directed therapy to MGZL occurring five years after curative cHL treatment, providing a practical reference for similar rare cases.

## Conclusion

MGZL may arise as a SMN after cHL and must be distinguished from relapse or PMBL. Repeat biopsy with comprehensive immunophenotyping and molecular profiling is essential for definitive diagnosis. Genomic-guided personalized therapy is pivotal to optimizing outcomes in patients with MGZL.

## Data Availability

The original contributions presented in the study are included in the article/supplementary material. Further inquiries can be directed to the corresponding author.
